# Impact of Tracheal Tube on Swallowing in Post-Operative Head and Neck Cancer Patients: Scintigraphic Analysis

**DOI:** 10.1007/s00455-020-10222-y

**Published:** 2020-12-05

**Authors:** Jacopo Galli, Maria Raffaella Marchese, Tiziana Di Cesare, Laura Tricarico, Giovanni Almadori, Valeria Tempesta, Venanzio Valenza, Gaetano Paludetti

**Affiliations:** 1 Department of Aging, Neuroscience, Orthopedics and Head and Neck Sciences, UOC of Otorhinolaryngology, Istituto di Otorinolaringoiatria “Fondazione Policlinico Universitario A. Gemelli IRCCS – Università Cattolica del Sacro Cuore”, Roma, Italy; 2 Department of Aging, Neuroscience, Orthopedics and Head and Neck Sciences, UOC of Otorhinolaryngology, Policlinico “A. Gemelli” Foundation, L.Go “A. Gemelli”, 8, 00168 Rome, Italy; 3 Department of Nuclear Medicine, “Fondazione Policlinico Universitario A. Gemelli IRCCS”, Roma, Italy

**Keywords:** Deglutition, Deglutition disorders, Tracheal tube, Dysphagia, Oropharyngoesophageal scintigraphy, Swallowing, Tracheostomy

## Abstract

Dysphagia is common in tracheostomized patients who underwent head and neck surgery for cancer treatment. The objective of this study was to evaluate, by means of oropharyngoesophageal scintigraphy (OPES), the impact of an occluded tracheal tube (TT) on swallowing in patients treated for head and neck cancer before hospital discharge, to provide further information to the benefit of out-patient care management. From October 2018 to November 2019, we enrolled 19 tracheostomized patients (6 females and 13 males; mean age 61 years) who underwent primary surgical resection of head and neck tumor and swallowing rehabilitation during hospitalization. All subjects underwent a double-standard OPES, one with occluded tracheal tube and the other without TT, with their tracheal stoma being closed directly by a plaster. For each study, we assessed and compared the following quantitative parameters: oral transit time (OTTsec), pharyngeal transit time (PTTsec), esophageal transit time (ETTsec), oral retention index (ORI%), pharyngeal retention index (PRI%), esophageal retention index (ERI%), and aspiration percentage (AP%). The mean values of OTT, PTT, ORI%, PRI%, and ERI% were abnormal during OPES both with TT and without TT and did not statistically differ between the two tests (*p* > 0.05). Aspiration was detected in 4 cases out of 19 (21.05%) cases during OPES with TT and in 4/19 (21.05%) cases without TT who showed a mean AP% of 11.4% and 11.5% respectively (*p* > 0.05). Patients with abnormal AP% (> 0%) during OPES with TT showed aspiration signs without TT. Our study showed that the mere presence of a closed tracheal tube does not impact significantly the oropharyngeal transit of bolus during swallowing. This result suggests the possibility to maintain a small-diameter occluded tracheal tube in place for the postsurgical management of head and neck cancer patients.

## Introduction

Dysphagia is a dysfunction that deeply impacts patients, especially during post-surgery recovery. In particular, it is the most common short-term and long-term sequela in subjects undergoing head and neck oncologic surgery [1]. In this regard, tracheostomy with placed tracheal tube represents a safety measure which allows better management of pulmonary secretions and access to remove aspirated material, limiting bronchopulmonary complications. At a time, when hospitalization is getting shorter and the swallowing rehabilitation training is mainly carried out in the out-patient setting, it is even more important to clarify the real impact of tracheal tube. Over the last twenty years, there have been conflicting results in the literature on whether the presence of a tracheal tube impacts swallowing functions [2]. Initially, the majority of authors agreed to identify the tracheal tube as a cause of aspiration based on several mechanisms, first of all the decrease of laryngeal elevation [2, 3]. Nevertheless, over time, new research has opened to different results. In 2010, Leder et al. [4] demonstrated the absence of a causal relationship between tracheotomy and aspiration status in 25 patients who underwent fiberoptic endoscopic evaluation of swallowing (FEES), and more recently Kang et al. [5] showed in a Videofluoroscopic swallow study (VFSS) that the swallowing parameters related to laryngeal elevation, pharyngeal constriction, and esophageal opening did not change significantly between patients with and without tracheostomy. However, the methods employed so far to analyze the same scope were heterogeneous: the study groups often included both patients evaluated immediately after the tracheostomy and patients with the tracheal stoma stabilized [5, 6]. Furthermore, the possible mechanisms hypothesized to explain the increased risk of aspiration, such as the decrease in laryngeal elevation, the desensitization of the larynx, and the loss of protective reflexes, were the same for both the studies on the effect of TT occlusion [7] and the ones on the presence of TT [2]. Although the presence of the tracheostomy tube has been associated in multiple studies with the increased incidence of aspiration [2, 3], the hypothesis that we want to validate is that the use of a small-diameter and capped tracheotomy tube in the post-operative period does not increase the incidence of aspiration and it does not impact negatively the recovery of swallowing functions. Our study aimed to evaluate the intraindividual changes of the main qualitative and quantitative parameters of oropharyngoesophageal scintigraphy (OPES) obtained in tracheostomized patients who underwent surgery for head and neck cancer at the end of swallowing rehabilitation, proximal to their hospital discharge, firstly with a small occluded tracheal tube and then without tracheal tube simply occluding the stoma with a plaster.

## Patients and Methods

From October 2018 to November 2019, at the ENT department of Fondazione Policlinico Universitario A. Gemelli, we carried out a survey to recruit patients who underwent elective tracheostomy during surgical resection of head and neck cancer. The inclusion criteria were the following: tracheal tube with inner diameter of 5 mm and outer diameter of 9.4 mm; tolerance to tracheal tube capping for at least 48 h without pathologic desaturation during continuous monitoring of SpO2; ability to swallow basing on bedside examination (GUSS score > 19) [8] and Fiberoptic Endoscopic Evaluation of Swallowing (FEES) assessment (Pooling-score < 5) [9]; ability to suck through a straw without oral leakage; age > 18 years. We excluded patients with nasal tube feeding, poor cognition or poor general condition, total laryngectomies or patients unable to maintain the orthostatic position, and patients with neurological or other non-tumor-related dysphagia.

The oropharyngoesophageal scintigraphy (OPES) was performed twice by a blinded nuclear radiologist with long-standing experience in this field, according to the standard technique for a qualitative and quantitative analysis of swallowing described in our previous studies [10–13]. Firstly, patients were tested with the tracheal tube (TT) closed in place. After 10 min of rest, they repeated the OPES without the tracheal tube and closing the tracheal stoma with a plaster. At the end of the examination, the subjects ingested 100 mL of water to eliminate the radiation dose still present at the pharyngeal or esophageal level. The following quantitative parameters, expressed as absolute values in seconds or percentage, were evaluated: *oral transit time* (OTT) [normal value (n.v). < 1 s] [14], the time required for the bolus to leave the oral cavity after swallowing; *pharyngeal transit time* (PTT) (n.v. < 1.20 s) [15], the time interval between the appearance and the disappearance of the bolus in the pharynx; *esophageal transit time* (ETT) (n.v. < 10 s) [14], the time required for the bolus to pass through the esophagus and into the stomach; oral retention index (ORI%) (n.v. < 5% 1 s after the swallow) [16]; *pharyngeal retention index* (PRI%), the residual amount of radioactivity in the pharynx 10 s after deglutition (n.v. < 5% after a swallow—frequently equal to 0%) [17]; *esophageal retention index* (ERI%), the residual amount of radioactivity in the esophagus 10 s after swallowing (n.v. < 10% 10 s after a swallow) [18]; *aspiration percentage* (AP%) was assessed when tracheobronchial aspiration was detected during visual inspection of the images [19]. The calculation of AP% and the normative data of each parameter were described in our previous works [10, 11]. A written informed consent was obtained from all the participants included in the study. Statistical analysis was performed using commercially available software (Excel–Microsoft Corporation, Redmond, Washington, USA). Continuously distributed data were summarized as the mean and median and categorical variables with frequencies and percentages. The Wilcoxon signed rank test and Student t test were used for non-parametric and parametric variables respectively, when appropriate. A p value less than 0.05 was considered as significant.

## Results

From a group of 24 patients, 5 out of 24 (20.8%) met the exclusion criteria because of cognitive impairment (2/5 cases), inability to maintain the orthostatic position (2/5 cases) and neurological dysphagia (1/5 case). 19/24 (79.2%) patients were enrolled in the study. 6/19 (31.5%) cases were females and 13/19 (68.5%) were males, with a mean age of 61 years (min. 30 – max. 80). All patients underwent elective tracheostomy, in order to secure the airway during the immediate post-operative period, in addition to primary surgical removal of the cancer by different approach. The tracheostomy was performed in 9/19 (47.4%) patients undergoing COMbined MAndibulectomy and Neck Dissection Operation (COMMANDO) and reconstruction of the defect with free flaps for tongue cancer, in 4/19 (21%) cases who underwent excision of retromolar trigone cancer with marginal mandibulectomy, in 3/19 (15.8%) during Open Partial Horizontal Laryngectomy (OPHL), in 2/19 (5.3%) cases who underwent orbital exenteration and total maxillectomy for maxillary sinus surgery, and in 1/19 (5.3%) undergoing total parotidectomy and partial mandibulectomy for parotid tumor. The oropharyngoesophageal scintigraphy (OPES) was performed after a mean of 17.6 days (min. 9 -max. 28) from the tracheostomy.

The median, mean, and standard deviation (±SD) of each parameter calculated after both OPES studies are shown in Table 1. The median and mean values of oral (OTT), pharyngeal (PTT) transit time, and oral (ORI%), pharyngeal (PRI%), and esophageal (ERI%) retention index were abnormal during OPES with occluded tracheal tube and without tracheal tube. On the other hand, the values regarding esophageal transit time (ETT) were normal at both tests. The statistical differences between the results of each parameter of OPES with tracheal tube (TT) and the ones with direct stomal occlusion were not significant (*p* > 0.05) (Table 1) except for the ERI% that increased significantly without TT. The distribution of patients based on the normal value for each OPES parameter obtained with tracheal tube did not change after the OPES without TT (*p* > 0.05) (Table 2). In 15/19 (78.9%) cases, the aspiration percentage (AP %) was 0 both with TT and without TT. In the remaining cases (4/19 – 21.05%), the mean AP% was 11.5% ± 2.1 and 11.03% ± 2 in presence of TT and without TT, respectively (*p* > 0.05). The patients with AP% > 0 with TT were the same that inhaled without TT.

**Table 1:** Mean, median, standard deviation (± SD), and the significance of statistical difference (*p*) between the results of each parameter obtained during oropharyngeal scintigraphy (OPES) with tracheal tube (TT) in comparison with the ones obtained during OPES without TT

	OPES with TT			OPES without TT			***p***
	Mean	Median	±SD	Mean	Median	± SD	
Oral transit time (OTT sec., n.v. < 1)	1.5	1.3	± 0.9	1.6	0.9	± 1.3	> 0.05
Pharyngeal transit time (PTT sec., v.n. < 1.2)	2.4	2.4	± 1.3	2.1	1.7	± 1.4	> 0.05
Esophageal transit time (ETT sec., n.v. < 10)	8.9	7.8	± 3.7	9.3	8.8	± 3.7	> 0.05
Oral retention index (ORI%, n.v. < 5)	17.8	12	± 15.1	22	14	± 21.9	> 0.05
Pharyngeal retention index (PRI%, n.v. < 5)	9.7	6	± 10.3	8.8	6	± 9.2	> 0.05
Esophageal retention index (ERI%, n.v. < 10)	10.2	9	± 8.1	13.8	10	± 13.1	< 0.05
Aspiration percentage (AP%, n.v. 0)	11.4	–	± 0.2	11.5	–	± 0.2	> 0.05

**Table 2:** Prevalence of patients with normal and abnormal results for each parameter of oropharyngoesophageal scintigraphy (OPES) with tracheal tube (TT) and without TT

	OPES with TT		OPES without TT		p*
	n. cases with normal results	n. cases with abnormal results	n. cases with normal results	n. cases with abnormal results	
Oral transit time (OTT sec.)	5 (26.3%)	14 (73.6%)	8 (42.1%)	11 (57.8%)	> 0.05
Pharyngeal transit time (PTT sec.)	4 (21.1%)	15 (78.9%)	6 (31.5%)	13 (68.4%)	> 0.05
Esophageal transit time (ETT sec.)	11 (57.8%)	8 (42.1%)	12 (63.1%)	7 (36.8%)	> 0.05
Oral retention index (ORI%)	2 (10.5%)	17 (89.4%)	1 (5.2%)	18 (94.7%)	> 0.05
Pharyngeal retention index (PRI%)	5 (26.3%)	14 (73.6%)	5 (26.3%)	14 (73.6%)	> 0.05
Esophageal retention index (ERI%)	9 (47.36%)	10 (52.63%)	7 (36.84%)	12 (63.15%)	> 0.05
Aspiration percentage (AP%)	15 (78.9%)	4 (21.0%)	15 (78.9%)	4 (21.0%)	> 0.05

*Statistical significance between the prevalence of normal and abnormal values obtained during OPES with TT and without TT

## Discussion

Swallowing dysfunction after tracheostomy has been widely debated in the last two decades. Prior researches showed that tracheostomy tubes could impair swallowing causing an increased risk of aspiration through the following mechanisms: decrease in laryngeal elevation due to an anchoring effect of the tracheal tube [2], desensitization of the larynx and loss of the protective reflex [20], and uncoordinated laryngeal closure attributable to chronic upper airway bypass [21]. On the contrary, recent investigations have come to the opposite conclusion [4, 5]. Despite the high number of studies, most of them were methodologically not homogeneous, lacking of a standardized methods for the swallowing assessment. In this regard, our study suggests that a small-diameter and capped tracheal tube does not interfere with swallowing. Most of our cases 15/19 (78.9%) did not inhale, and the subjects 4/19 (21.05%) who aspirated during OPES with TT showed signs of aspiration also without TT and with the tracheal stoma directly occluded. However, the limitations of this study, including small sample size, do not allow to strongly affirm that aspiration is independent of tracheal tube; future studies are needed in this regard. We designed the study starting from few strong evidences. First of all, it is widely known that aspiration occurs more frequently when the tracheostomy tube is open [22] due to the inability to increase subglottic pressure in this condition, which may be responsible for increased aspiration [23]. Second, most authors agree that the presence of a cuff or the presence of a tracheal tube with inadequate diameter can cause dysphagia, suggesting that rehabilitation consequently involves the removal of the cuff and the progressive reduction in tracheal tube diameter [24]. For these reasons, we assessed the effect of the tracheal tube in a closed respiratory system (*i.e.*, closed tracheal tube or closed tracheal stoma). Recent studies [4, 5] did not standardize the sample based on the tracheal tube features (diameter and cuff), thus providing biased results. The effect of tracheal tube on swallowing was previously studied by means of videofluoroscopy (VFS) [5] and FEES [4, 6]. Even though VFS is still considered the gold standard, there are numerous reports in literature that emphasize the validity of OPES for the swallowing assessment [12, 25]. In clinical practice, OPES is the only tool available to provide an exact percentage of the aspiration into the respiratory tract [26] and an exact measurement of how long each of the three swallowing stages take, with detailed calculation of transit time and of any bolus retained in the oral cavity, pharynx or esophagus. The main limitation of this technique is the lack of well-defined morphological details of the structures involved [27, 28]. However, the measurements of premature pharyngeal entry, pharyngeal transit time, and post-swallow pharyngeal stasis by scintigraphy are well correlated with those of VFS [29]. In a recent study, Fattori B. et al. [30] compared FEES, VFS, and OPES concluding that all three are capable of supplying an accurate diagnosis of oropharyngeal dysphagia and, more specifically that the detection of airway aspiration is recognized as the foremost advantage of scintigraphy. We selected OPES also because it is well tolerated by patients, and it could be repeated for treatment planning, rehabilitation, and follow-up of patients thanks to the lower dosage of radiation (0.043 Gy and 0.011 mSv/MBq) [31]. On the other hand, the OPES technique forced us to exclude from the sample seriously impaired patients unable to suck through a straw or to maintain the erect position, which is another important limit of our work since the inclusion of more severe dysphagic patients may have increased the incidence of aspiration.

Our study aimed to clarify the real impact of tracheal tube on swallowing. In general, patients who underwent major head neck oncologic surgery start the rehabilitation within few days after surgery, and the tracheostomy and tracheal tube are undoubtedly conditions that could ensure better management of pulmonary secretions allowing access to remove aspirated material, therefore, reducing bronchopulmonary complications during the swallowing training. This is even more important for two reasons: first, because the duration of hospitalization has shortened over time, and for this reason, the swallowing rehabilitation is mainly carried out on an out-patient basis. Second, in the cases who require adjuvant radiotherapy, the maintenance of the tracheostomy is an indispensable safety condition.

Discharging patients from a hospital with a tracheal tube in place is extremely demanding for both the patients and their caregivers mainly due to the greater attention required in the management of the patient at home. These undoubtful negative effects are outweighed by the benefit of the tracheal tube to assist in maintaining pulmonary health considering that pneumonia is one of the main complications in patients with oropharyngeal dysphagia [32].

In 2007, Coscarelli et al. [33] described the results of a new swallowing rehabilitation protocol, specifically started two weeks after surgery without the tracheal tube, applied in 33 patients who underwent open partial horizontal laryngectomy (OPHL). The time for the complete recovery of oral intake and the incidence of pneumonia were the selected outcomes. They concluded that during rehabilitation exercises, the absence of tracheal tube improves swallowing by a better closure of the remaining larynx. Nevertheless, this study lacked the control group and did not provide objective data of the swallowing performances. Similarly to our results, Leder et al. [34] investigated by means of VFS the effects of tracheal tube on aspiration status, interpreting the modified barium swallowing by repeated viewings of the videotapes in order to confirm the presence or absence of aspiration in patients with head and neck cancer after two weeks from the surgery, and they did not find any differences in aspiration status of patients with and without tracheal tube. Regarding the abnormal OPES data obtained from oral and pharyngeal phases’ analysis in our group, they seem to be related with the type of surgery that extensively involved oral and oropharyngeal sites, disrupting the dynamic of swallowing, without impacting significantly on the main goal of our study.

In conclusion, our overall results demonstrated the lack of statistically significant differences in each of the oropharyngoesophageal scintigraphy (OPES) parameters in the two conditions of the study, with and without the use of a small, closed tracheal tube, minimizing the impact of the tracheal tube on swallowing in head and neck cancer patients.
